# The Yin and Yang of regulatory T cells in immunotherapy

**DOI:** 10.18632/oncotarget.24394

**Published:** 2018-02-03

**Authors:** Yan Li, James P. Di Santo

**Affiliations:** James P. Di Santo: Innate Immunity Unit, Institut Pasteur, Paris, France; Inserm, Paris, France

**Keywords:** humanized mice, cancer immunotherapy, regulatory T cells, IL-2 induced toxicity

In the recent decades, there has been a growing interest in harnessing the power of host immunity to fight against cancer. Advances in our understanding of immune mechanisms of tumor surveillance have dramatically improved the efficacy of immunotherapies. Nevertheless, severe toxicity becomes a prominent and shared ‘feature’ among many anti-tumor strategies. Administration of cytokines, such as IL-2, to patients represents one landmark in the development of cancer immunotherapies [1]. Remarkably, IL-2 therapy was shown to induce complete durable regressions in some cases of metastatic melanoma and renal carcinoma, prompting the U.S. Food and Drug Administration to approve high-dose IL-2 as an immunotherapy in 1992. While considerable progress has been made to increase the response rate to IL-2 therapy, severe toxicities limit IL-2 therapy to pre-selected ‘healthy’ patients.

To better understand the mechanisms underlying toxicity associated with IL-2 immunotherapy, we employed a human immune system (HIS) mouse model [2]. In HIS mice, multi-lineage human immune subsets are stably reconstituted following transfer of human hematopoietic stem cells to immunodeficient BALB/c *Rag2*^−/−^*Il2rg*^−/−^*Sirpa*^NOD^ (BRGS) recipients. We found that dose-dependent morbidity and mortality of IL-2 therapy can be provoked in HIS mice after hydrodynamic injection of IL-2 encoding plasmids, but not in BRGS mice without human cells. This allowed us to dissect the contribution of individual human immune cells to IL-2 mediated toxicity *in vivo*. We showed that depletion of human T cells abolished toxicity, thus pointing to a central role of T cells in IL-2 mediated toxicity. Unexpectedly, we observed decreased percentages and function of regulatory T cells (T_reg_) in HIS mice after high-dose IL-2 therapy. This suggested that T_reg_ dysfunction might also be involved in IL-2 toxicity resulting in uncontrolled activation and proliferation of effector T cells (T_eff_). To test this hypothesis, we assessed whether depletion of T_reg_ or blocking of T_reg_ function would cause severe toxicity in HIS mice under low-dose IL-2 therapy, which is well tolerated in both patients and in HIS mice. In accordance with our hypothesis, interfering with T_reg_ function in low-dose IL-2 HIS mice provoked clinical and pathological signatures of high-dose IL-2 toxicity. In contrast, preserving T_reg_ function using the PIM-1 kinase inhibitor Kaempferol could ameliorate the toxicity associated with high-dose IL-2. Through modeling IL-2 toxicity in HIS mice, we have discovered a novel role for T_reg_ in modulating cytokine-mediated toxicity and challenge the prevalent notion that T_reg_ are detrimental for immunotherapy.

T_reg_ are present in diverse tumor types and high ratios of tumor infiltrating T_reg_ to CD8 T cells are correlated with poor prognosis [3]. For patients with metastatic melanoma, high frequencies of circulating ICOS^+^ T_reg_ predict poor response from high-dose IL-2 therapy [4]. Based on the evidence that T_reg_ dampens anti-tumor responses, novel optimized IL-2 therapies aim to limit T_reg_ stimulation using mutant IL-2 molecules or by coupling IL-2 with antibodies [5, 6]. These new IL-2 therapies favor expansion of T_eff_ over T_reg_ with the hope to augment antitumor responses. Our study, on the other hand, illustrates the essential role of T_reg_ in taming the serious autoimmunity associated with IL-2 administration. This finding should be considered when designing new strategies to improve the efficacy of immunotherapies. As tumor antigens are normal or mutated self-antigens, effective antitumor immunity often means breaking self-tolerance systemically or locally. Whether autoimmunity due to the loss of T_reg_ suppression in IL-2 therapy directly promote antitumor efficacy remains to be explored.

Immune checkpoint blockade, such as CTLA-4 and PD-1 antibodies, can provoke treatment-limiting side effects. CTLA-4 is a potent co-inhibitory marker expressed by T_reg_ as well as activated T_eff_ cells. The mechanism of CTLA-4 antibody was initially believed to be a blockade of CTLA-4 function on both T_eff_ and T_reg_, but was later shown to be due to T_reg_ depletion [7]; a finding that we confirmed in HIS mice. Together, these studies demonstrate the value of HIS mice to help elucidate immune mechanisms associated with anti-cancer therapies. As such, HIS mice can be used as a preclinical platform to assess the efficacy and toxicity of novel checkpoint immunotherapies or combinational therapies for cancer.
